# Escalated regeneration in sciatic nerve crush injury by the combined therapy of human amniotic fluid mesenchymal stem cells and fermented soybean extracts, Natto

**DOI:** 10.1186/1423-0127-16-75

**Published:** 2009-08-23

**Authors:** Hung-Chuan Pan, Dar-Yu Yang, Shu-Peng Ho, Meei-Ling Sheu, Chung-Jung Chen, Shiaw-Min Hwang, Ming-Hong Chang, Fu-Chou Cheng

**Affiliations:** 1 Department of Neurosurgery, Taichung Veterans General Hospital, 407 Taichung, Taiwan, Republic of China; 2 Stem Cell Center, Department of Medical Research, Taichung Veterans General Hospital, 407 Taichung, Taiwan, Republic of China; 3 Department of Neurology, Taichung Veterans General Hospital, 407 Taichung, Taiwan, Republic of China; 4 Department of Neurosurgery, Chang Bing Show Chwan Memorial Hospital, 505 Changhua, Taiwan, Republic of China; 5 Department of Veterinary Medicine, Institute of Medical Technology, National Chung-Hsing University, 402, Taichung, Taiwan, Republic of China; 6 Bioresource Collection and Research Center, Food Industry Research and Development Institute, 300 Hsinchu, Taiwan, Republic of China

## Abstract

Attenuation of inflammatory cell deposits and associated cytokines prevented the apoptosis of transplanted stem cells in a sciatic nerve crush injury model. Suppression of inflammatory cytokines by fermented soybean extracts (Natto) was also beneficial to nerve regeneration. In this study, the effect of Natto on transplanted human amniotic fluid mesenchymal stem cells (AFS) was evaluated. Peripheral nerve injury was induced in SD rats by crushing a sciatic nerve using a vessel clamp. Animals were categorized into four groups: Group I: no treatment; Group II: fed with Natto (16 mg/day for 7 consecutive days); Group III: AFS embedded in fibrin glue; Group IV: Combination of group II and III therapy. Transplanted AFS and Schwann cell apoptosis, inflammatory cell deposits and associated cytokines, motor function, and nerve regeneration were evaluated 7 or 28 days after injury. The deterioration of neurological function was attenuated by AFS, Natto, or the combined therapy. The combined therapy caused the most significantly beneficial effects. Administration of Natto suppressed the inflammatory responses and correlated with decreased AFS and Schwann cell apoptosis. The decreased AFS apoptosis was in line with neurological improvement such as expression of early regeneration marker of neurofilament and late markers of S-100 and decreased vacuole formation. Administration of either AFS, or Natto, or combined therapy augmented the nerve regeneration. In conclusion, administration of Natto may rescue the AFS and Schwann cells from apoptosis by suppressing the macrophage deposits, associated inflammatory cytokines, and fibrin deposits.

## Introduction

Several approaches have been proposed to have beneficial effects on peripheral nerve regeneration, including application of an electric field, transplantation of stem cells, and administration of neurotrophic factors [1-4]. The implantation of embryonic stem cells, neural stem cells, and mesenchymal stem cells has been shown to exert beneficial effects on peripheral nerve regeneration. Cell replacement, trophic factor production, extracellular matrix molecule synthesis, guidance, remyelination, microenvironmental stabilization, and immune modulation have recently been proposed as beneficial mechanisms after cell implantation [1,5,6]

Recent evidence has shown amniotic fluid to be a novel source of stem cells for therapeutic transplantation. Amniotic fluid-derived stem cells express characteristics of both mesenchymal and neural stem cells [7]. In our previous work, we demonstrated that transplantation of amniotic fluid mesenchymal stem cells (AFS) promoted peripheral nerve regeneration [2,3]. Furthermore, increased implanted stem cell survival was augmented by the suppression of inflammatory cytokines through the inhibition of inflammatory cell deposits [8,9]. Thus the modulation of inflammatory response could attenuate the apoptotic cascade of the transplanted stem cells, which implicates a significant improvement in nerve regeneration.

After sciatic nerve injury, fibrin is deposited at the nerve and its deposition exacerbates nerve damage [10]. Fibrin clearance correlates with regeneration, while fibrin deposition delays nerve regeneration by arresting Schwann cells in a proliferating and non-myelinating state [11]. In addition, fibrin deposited in the sciatic nerve after injury changes the composition of extracellular matrix and inhibits Schwann cell migration [12]. In contrast, inhibition of fibrin deposition reduces macrophage adhesion and decreases cytokine production such as IL-1 and TNF-α, which is in parallel with nerve regeneration [13-16]. Fermented soybeans extracts (Natto), which form part of the traditional Japanese diet, promote fibrinolytic activity in the circulation in a similar manner to oral urokinase [17-20]. In our previous investigation, oral administration of Natto rescued the Schwann cell apoptosis by inhibition of fibrin deposits and suppression of inflammatory cytokines, which was in line with restoration of extracellular matrix and increased nerve myelination [21].

Therefore, the present study was designed to evaluate whether the combination of Natto and AFS transplantation could synergically augment the peripheral nerve regeneration. The potential contribution of the anti-apoptotic and anti-inflammatory effects of Natto was also investigated.

## Materials and methods

### Animal model

Sprague-Dawley rats weighing from 250-300 g were used in this study; permission was obtained from the Ethics Committee of Taichung Veterans General Hospital for their use. The rats were anesthetized with 4% isoflurane in induction followed by a maintenance dose (1%-2%) [2% (v/v %) in 70% N_2_O/30% O_2_; 0.5 l/min flow rate] (A.S.D.1000). The left sciatic nerve was exposed under a microscope using the gluteal muscle splitting method. A vessel clamp (B-3, pressure 1.5 gm/mm^2^, S&T Marketing LTD, Switzerland) was applied 10 mm from the internal obturator canal for 20 min [2]. The crush site was then sutured with 9-0 nylon over the epineuria as a mark. The animals were categorized into four groups. In group I, the left sciatic nerve was crushed and wrapped with fibrin glue. The animals were fed with normal saline through oral gastric tube (OG) for 7 consecutive days. In group II, the left sciatic nerve was crushed and wrapped with fibrin glue. The animals were fed with Natto extract (Synmax, Taipei, Taiwan) 16 mg/day (regular dosage for humans adjusted with body weight for rats) in 0.5 ml normal saline through oral gastric tube (O-G) feeding for 7 consecutive days [21]. In group III, AFS embedded in fibrin glue was delivered to the injured nerve. The animals were fed with normal saline through O-G feeding for 7 consecutive days. In Group IV, AFS embedded in fibrin glue was delivered to the injured nerve. The animals were fed with Natto (Synmax, Taipei, Taiwan) 16 mg/day in 0.5 ml normal saline through oral gastric tube (O-G) feeding for 7 consecutive days. All animals received rehabilitation therapy on a metal mesh every week. Food and water were provided *ad libitum *before and after the experiments. The animals were kept in a temperature-controlled environment of 37°C, and were exposed to alternate light and dark periods of 12 h. All animals were treated and cared for in accordance with the guidelines recommended by the Ethics Committee of Taichung Veterans General Hospital.

### Preparation and culture of human amniotic mesenchymal stem cells (AFS)

Amniotic fluid samples (20 ml) were obtained by amniocentesis performed between 16 and 20 weeks of gestation for fetal karyotyping. For culturing amniocytes, four primary *in situ *cultures were set up in 35 mm tissue culture-grade dishes using Chang medium (Irvine Scientific, Santa Ana, CA). Microscopic analysis of Giemsa-stained chromosome banding was performed, and the rules for metaphase selection and colony definition were based on the basic requirements for prenatal cytogenetic diagnosis in amniocytes (Moertel CA 1992). For culturing AFS, non-adhering amniotic fluid cells in supernatant medium were collected on the fifth day after primary amniocytes culture and maintained until completion of fetal chromosome analysis. The cells were then centrifuged and plated in 5 ml of β-minimum essential medium (β-MEM; Gibco-BRL) supplemented with 20% fetal bovine serum (FBS; Hyclone, Logan, UT, USA) and 4 ng/ml basic fibroblast growth factor (bFGF; R&D Systems, Minneapolis, MN, USA) in a 25 cm^2 ^flask and incubated at 37°C with 5% humidified CO_2_[2]. This protocol (950203/C06022) was approved by the Institutional Review Board (IRB) of Taichung Veterans General Hospital and written informed consent was obtained from all patients.

### Grafting procedure

AFS were labeled with Hoechst 33342 before grafting. A volume of 25 μl of AFS with a total amount of 10^6 ^cells was suspended in 25 μl of Fibrin glue (Aventis Behring, Germany) containing the woven Surgicel (Johnson & Johnson, USA) and transplanted into the injured site immediately after crush [2].

### Analysis of functional recovery

A technical assistant who was blinded to treatment allocation evaluated sciatic nerve function before, 1 day and then weekly after the surgery. The evaluation method included ankle kinematics and sciatic function index (SFI) [2,3]. In the sagittal plane analysis, the following formula was used in the mechanical analysis of rat ankle: θ ankle = θ foot- leg 90. Several measurements were taken from the footprint by red ink print: (i) distance from the heel to the third toe, the print length (PL); (ii) distance from the first to fifth toe, the toe spread (TS); and (iii) distance from the second to the fourth toe, the intermediary toe spread (ITS). All three measurements were taken from the experimental (E) and normal (N) sides. The SFI was calculated according to the equation:



The SFI oscillates around 0 for normal nerve function, whereas SFI around -100 represents total dysfunction.

### Electrophysiological study

Six left sciatic nerves from individual groups were exposed 4 weeks after operation. Electric stimulation was applied to the proximal side of the injured site; the conduction latency, and the compound muscle action potential (CMAP) were recorded with an active electrode needle 10 mm below the tibia tubercle and a reference needle 20 mm from the active electrode. The mean length from the stimulation from the active recording electrode was 52.4 ± 0.4 mm. The stimulation intensity and filtration ranges were 5 mA and 20-2000 Hz, respectively. The CMAP data and conduction latency were converted to ratios of the injured side divided by the normal side to adjust for the effect of anesthesia [2,3].

### Quantification of pro-inflammatory cytokines

Six nerve tissues in each group for every single parameter were removed 7 days after the operation. The regenerating tissues (10 mm in length, marked "crush site" in the middle of the nerve) were retrieved and the samples were stored at -80°C. Subsequently, each tissue sample was homogenized with Laemmli SDS buffer. The homogenate was centrifuged for 10 minutes at 12000 g at 4°C. The tissue homogenate, 100 μl in triplicate, was applied to a microtiter plate and allowed to adhere overnight at 4°C. The microtiter plates were washed with phosphate-buffered saline (PBS)-Tween-20 and blocked with 1% BSA in PBS (200 μl) for 1 h. The plates were then treated with respective primary antibodies and allowed to set for 6 hours at 37°C. One hundred μl of the respective polyclonal antibodies against TNF-α and IL-1β (R&D Systems, Inc.) were applied overnight to microtiter plates. After further washing in PBS-Tween-20, the plates were incubated with the respective second antibody conjugate to alkaline phosphate (100 μl) for 1 h. The reaction was developed using p-nitrophenyl phosphate, disodium (3 mM) in carbonate buffer, pH 9.6 (100 mM Na_2_CO_3 _and 5 mM MgCl_2 _(150 μl), and the reaction was terminated after 30 minutes using 0.5 N NaOH (50 μl). The absorbance of colored product was read at 450 nm using a microplate reader (Bio-Tek Instruments). The relative amount of antigen present was measured from the densitometric reading against a standard curve.

### Terminal deoxynucleotidyl transferase-mediated biotinylated dUTP nick-end labeling (TUNEL) assay

Serial 8 μm-thick sections of sciatic nerve (7 days after surgery) were cut on a cryostat and mounted on superfrost/plus slides (Menzel-Glaser, Braunschweig, Germany). The TUNEL assay (Roche Molecular Biochemicals, Mannheim, Germany) was carried out as previously described [22]. Apoptotic cells were defined as those cells with TUNEL-positive nuclei that were condensed and fragmented, as assayed by DAPI (Molecular Probes, Eugene, OR; 1:2,000 dilutions) or Hoechst 33342. The number of apoptotic transplanted cells is expressed as a percentage of the total number of nuclei counted, with at least 25,000 nuclei for each condition.

### Immunohistochemistry

Serial 8 μm-thick sections of sciatic nerve were cut on a cryostat, mounted on superfrost/plus slides (Menzel-Glaser, Braunschweig, Germany) and were subjected to immunohistochemistry with antibodies against CD68 (Chemicon, 1:200 dilution), CD 8 (Serotec 1:200 dilution), CD19 (Thermo, 1:200 dilution), neutrophil (Abcam, 1:200 dilution), goat anti-human fibrin (1:500; Chemicon, 1:500), neurofilament (Chemicon, 1:300 dilution) (7 days after surgery), and S-100 (Neomarkers, 1:400 dilution) (4 weeks after surgery) for the detection of inflammatory cells, Schwann cells, and nerve fibers, respectively. The immunoreactive signals were visualized by goat anti-mouse IgG (FITC) (Jackson, 1:200 dilution), anti-mouse IgG (Rhodamine) (Jackson, 1:200 dilution), or 3, 3'-diaminobenzidine brown color. Among longitudinal consecutive resections, five consecutive resections contiguous to a maximum diameter were chosen to be measured. Of 100 squares with a surface area of 0.01 mm^2 ^each, 20 were randomly selected in an ocular grid and used to count the number of inflammatory cells. For the determination of neurofilament and S-100, six nerves in each group were cut longitudinally into 8 μm-thick sections and stained with each antibody. The maximum diameter of the resected nerve tissue with crush mark was chosen to be examined. Areas of activities (0.2 mm^2^) appeared as density against the background and were measured by a computer image analysis system (Alpha Innotech Corporation, IS 1000).

### Histological examination

After the neurobehavioral and electrophysiological testing, six rats in each group underwent transcardial perfusion with 4% paraformaldehyde 0.1 mol phosphate buffer (pH 7.4) after being reanesthetized. The sciatic nerve was harvested from the animals after the electrophysiological testing, and the nerve tissue was fixed on a plastic plate by stay sutures to keep the nerve straight [2]. The bilateral gastrocnemus muscle was harvested from each animal. The ratio of muscle weight (left/right) was recorded for analysis. The nerve was embedded, cut longitudinally into sections 8 μm thick and stained with haematoxylin-eosin (H&E) for the measurement of vacuole number and nerve integration. Among longitudinal consecutive resections, five consecutive resections contiguous to a maximum diameter were chosen and used to collect the data for comparison. Of 100 squares with a surface area of 0.01 mm^2 ^each, 20 were randomly selected in an ocular grid and used to count the vacuole number.

### Statistical analysis

Data are expressed as the mean ± SE (standard error). The statistical significance of differences between groups was determined by one-way analysis of variance (ANOVA) followed by Dunnett's test. In SFI and angle of ankle study, the results were analyzed by repeated-measurement of ANOVA followed by the multiple comparison method of Bonferroni. A *p *value less than 0.05 was considered significant.

## Results

### AFS+Natto administration significantly escalated motor and electrophysiological functions

**Figure 1:**
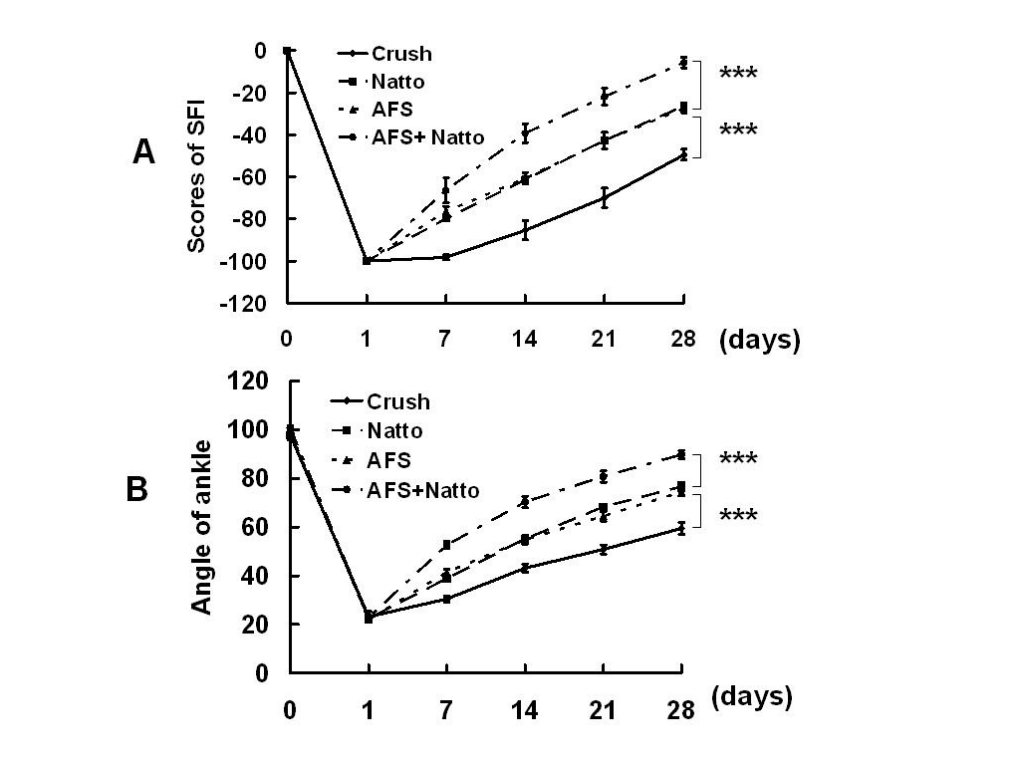
**Neurobehavioral evaluation**. A representative illustration of SFI (A) and angle of ankle (B) in the four treatment groups is shown. *** p < 0.001, n = 6.

SFI was significantly different among the individual groups (p < 0.001). SFI was escalated significantly in groups II, III, and IV as compared with group I (p < 0.001, p < 0.001, and p < 0.001, respectively). In addition, a significant discrepancy also existed between groups IV and III and IV and II (p < 0.01 and p < 0.01, respectively). However, no significant difference existed between groups II and III (Figure 1A). The presentation of angle of ankle also showed the same trends (p < 0.001). Angle of ankle was escalated significantly in groups II, III, and IV as compared with group I (p < 0.01, p < 0.01, and p < 0.01, respectively). A significant discrepancy also existed between groups IV and III and IV and II (p < 0.01 and p < 0.01, respectively). However, no significant difference existed between groups II and III (Figure 1B).

**Figure 2:**
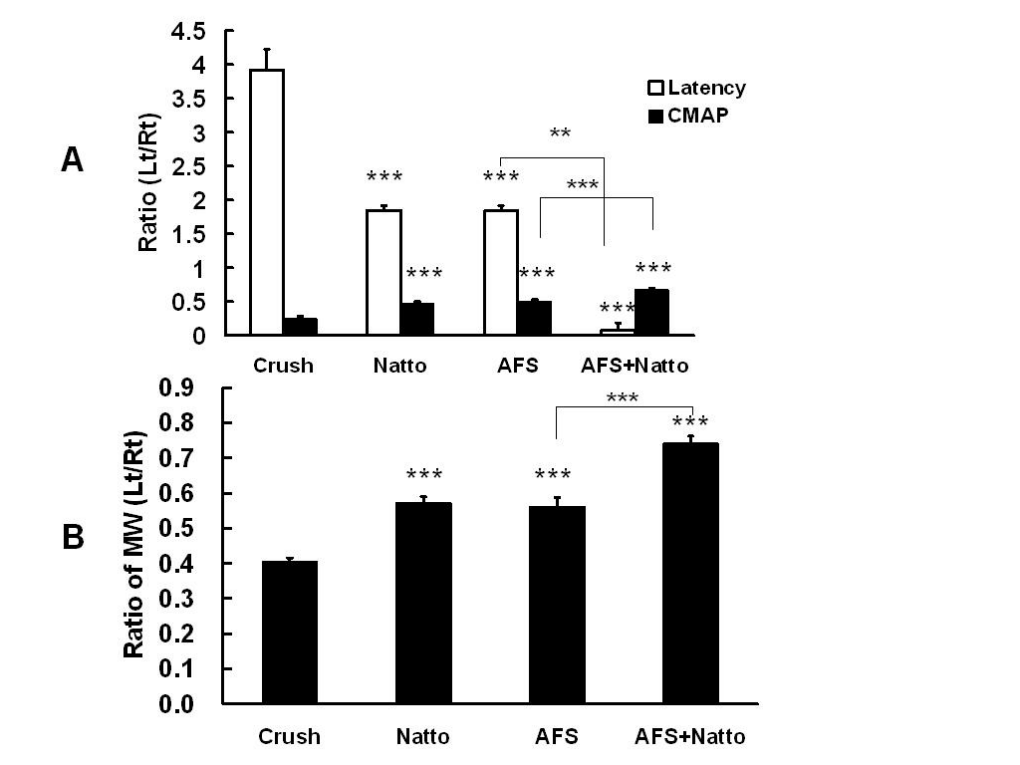
**Electrophysiological studies and gastrocnemus muscle weight**. The ratios of CMAP and conduction latency (A) and the ratio of the weight of the gastrocnemus muscles (B) were determined 4 weeks after injury in the four individual groups. P values (*** p < 0.001) in the Natto, AFS, and Natto+ AFS groups are determined relative to the crush group. P value (** p < 0.01) in the Natto+ AFS group indicates relative to the AFS group, n = 6.

The average ratio of CMAP in the four different groups was 0.25 ± 0.04% (group I), 0.47 ± 0.03% (group II), 0.51 ± 0.02% (group III), and 0.68 ± 0.02% (group IV), respectively (Figure 2A). There were significant differences between groups I and II (p < 0.001), I and III (p < 0.001), I and IV (p < 0.001), II and IV (p < 0.001), and III and IV (p < 0.001), respectively. However, no significant difference existed between groups II and III (p = 0.28).

The average ratio of conduction latency in the four different groups was 3.92 ± 0.31% (group I), 1.85 ± 0.07% (group II), 1.84 ± 0.08% (group III), and 1.38 ± 0.11% (group IV), respectively (Figure 2A). There were significant differences between groups I and II (p < 0.001), I and III (p < 0.001), I and IV (p < 0.001), II and IV (p = 0.003), and III and IV (p = 0.005), respectively. However, no significant difference existed between groups II and III (p = 0.89).

The average ratio of gastrocnemus muscle weight (left/right) in the four different groups was 4.0 ± 1.2% (group I), 5.7 ± 2.0% (group II), 5.6 ± 2.8% (group III), and 7.4 ± 2.2% (group IV), respectively (Figure 2B). The significant difference showed the same trend as that in the ratio of CMAP and in the ratio of conduction latency.

The above findings reveal that either AFS or Natto treatment alone promoted nerve regeneration better than the control. However, the combined treatment of AFS and Natto showed the most beneficial effects.

### AFS+Natto administration promoted early and late nerve regeneration

**Figure 3:**
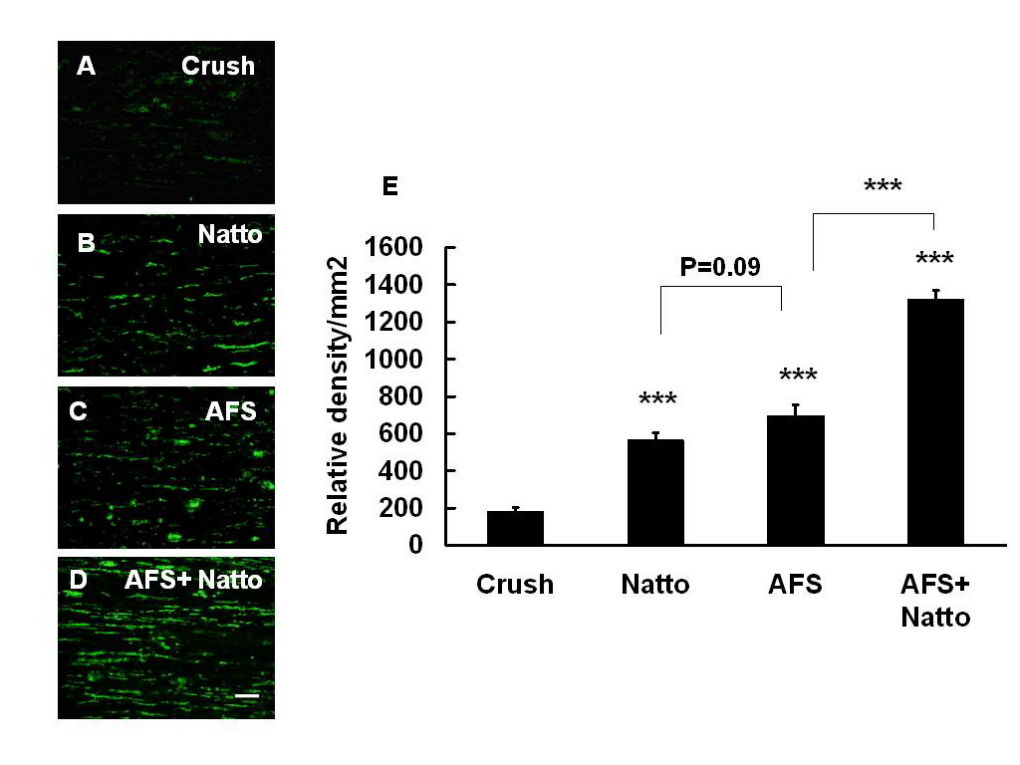
**Determination of neurofilament**. The nerve tissues were retrieved 7 days after injury and were subjected to IHC with antibody against neurofilament in the four treatment groups: (A) Crush, (B) Natto, (C) AFS, and (D) AFS+ Natto. The quantitative analysis of relative density of neurofilament is depicted in (E). P values in the Natto, AFS, and Natto+ AFS groups were determined relative to the crush group. *** p < 0.001; n = 6; bar length = 50 μm.

Treatment with either Natto (974.83 ± 32.53 relative density/mm^2^) or AFS (863.33 ± 40.22 relative density/mm^2^) alone significantly enhanced expression of neurofilament as compared to non-treatment (211.3 ± 12.01 relative density/mms^2^) (p < 0.001 and p < 0.001, respectively). Furthermore, treatment with AFS+ Natto (127833 ± 30.57 relative density/mm^2^) produced a higher expression than either AFS (p < 0.001) or Natto (p < 0.001) alone (Fig. 3).

**Figure 4:**
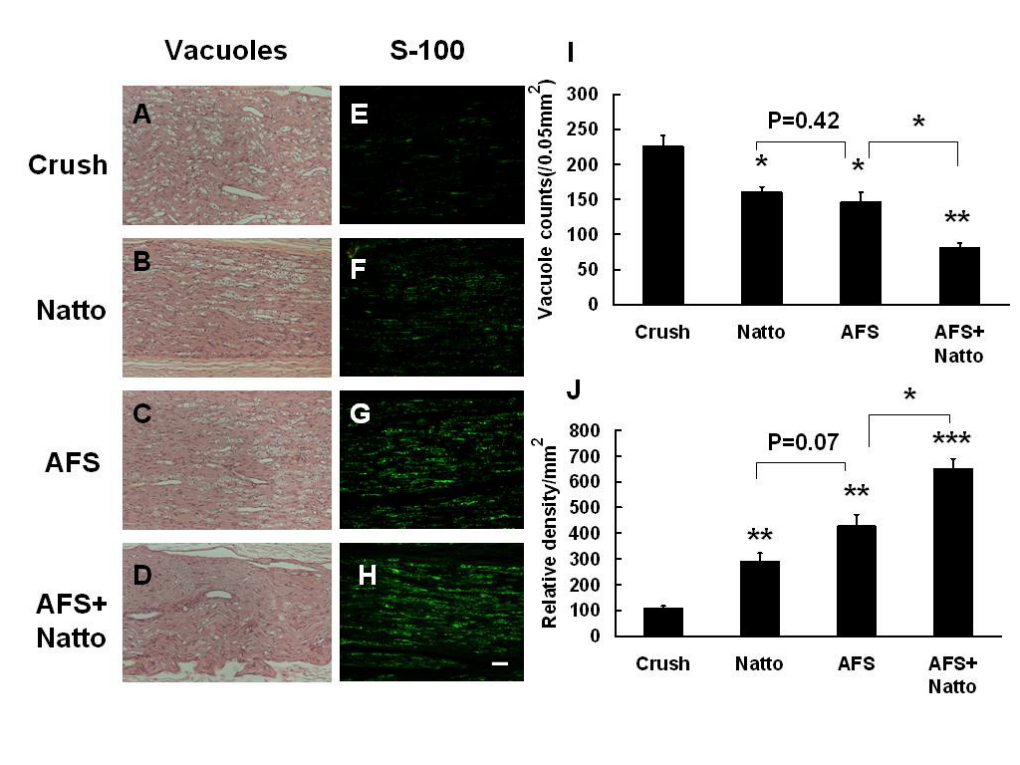
**Representative illustration of vacuole counts and S-100**. The left sciatic nerve was crushed, and the four different treatments were applied. After 4 weeks, the injured nerves were retrieved and were subjected to histological assay and antibody against S-100 for determination of vacuole counts (A-D) and myelination (E-F). Quantitative analysis is depicted in (I, J). * p < 0.05, ** p < 0.01, *** p < 0.001, n = 6, bar length = 50 μm.

The parameters of late nerve regeneration such as vacuole number and myelination as evidenced by the expression of S-100 are presented in Figure 4. Crush induced vacuole formation, and this event was reduced by the administration of Natto (p < 0.01) and AFS (p < 0.01). The combined treatment escalated the results (p < 0.001). Crush induced less expression of S-100, and a significant amount of S-100 expression was observed in the Natto- (p < 0.01) and AFS-treated groups (p < 0.01). The most beneficial restoration was observed in the combined treatment group (p < 0.001). Based on the early expression of neurofilament and late regeneration markers, treatment with either Natto or AFS alone promoted greater nerve regeneration than non-treatment; however, the combined treatment promoted significantly more regeneration than either of the single treatments.

### Natto administration reduced AFS apoptosis

**Figure 5:**
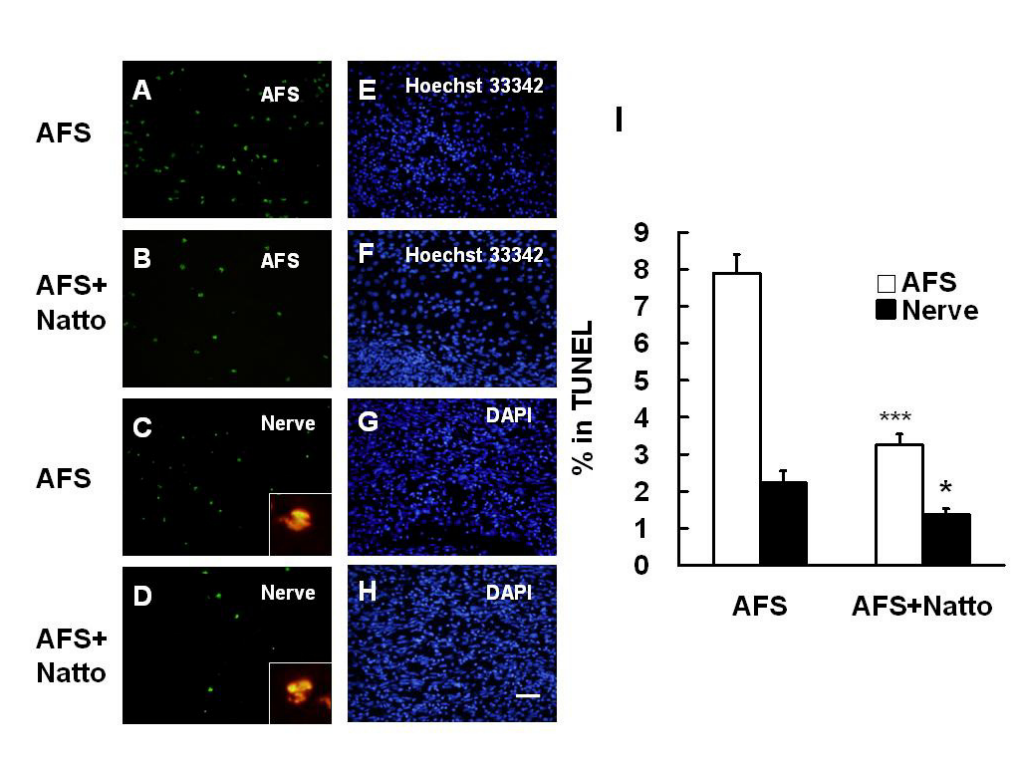
**Determination of apoptosis**. The nerve tissues implanted with AFS and treated with or without Natto were obtained 7 days after injury and subjected to apoptotic assay by TUNEL in the AFS (A-B) and crushed nerve (C-D) groups. (E, F) Hoechst 33342 staining in AFS corresponded to the areas in (A) and (B). (G, H) DAPI staining in the injured nerve corresponded to areas in (C) and (D). Magnification box indicated the merged imaging of TUNEL with S-100 (red). The quantitative analysis of TUNEL in the AFS and injured nerve groups is shown in (I). * p < 0.05, *** p < 0.001, bar length = 50 μm, n = 6.

Hoechst 33342-positive implanted AFS were found in the retrieved nerve tissues 7 days after grafting. Apoptotic AFS (7.9 ± 0.5%) were detected by the TUNEL-positive nuclei. The apoptosis of implanted AFS (3.25 ± 0.3%) was attenuated by Natto treatment (p < 0.001) (Fig. 5A, B). These findings indicate that one of the beneficial effects of Natto may be to strengthen the viability of implanted AFS so as to prevent apoptosis. Furthermore, the reduced apoptosis in Schwann cells distributed in the crushed nerve was in line with the attenuation of AFS apoptosis from 2.23 ± 0.32% to 1.37 ± 0.16% (p < 0.001) (Figure 5C, D).

### Natto administration attenuated inflammatory response and fibrin deposition, contributing to significant nerve regeneration

**Figure 6:**
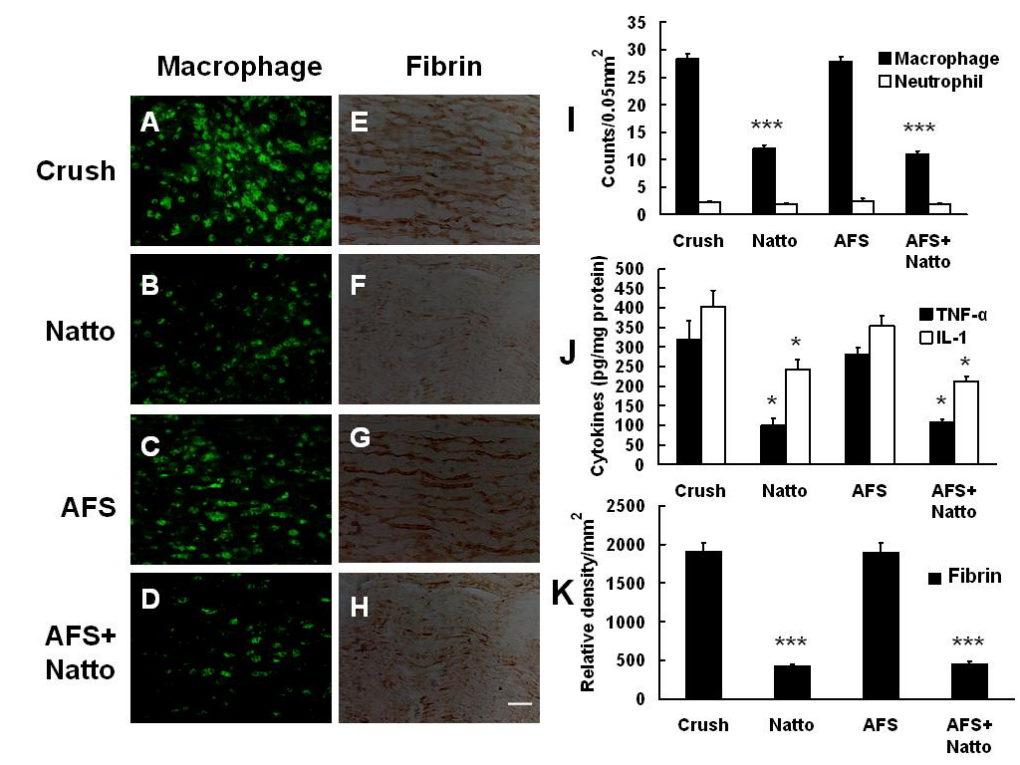
**Determination of inflammatory cells and associated cytokines**. The nerve tissues were retrieved 7 days after injury and were subjected to IHC with antibodies against CD68, neutrophils, fibrin and ELISA studies of pro-inflammatory cytokines. The representative samples of deposits of macrophages (A-D) and neutrophils (E-H) in injured nerve are shown. The quantitative analysis of macrophages and neutrophils (I), inflammatory cytokines (J), and density of fibrin aggregation (K) is depicted. * p < 0.05, *** p < 0.001. bar length = 50 μm, n = 6.

Over-activated inflammatory response is a detrimental stress on the nerve tissues and is a potential cytotoxic factor in the survival of implanted cells. Macrophages play a pathogenic role in peripheral nerves Clearance of macrophage deposits and inhibition of inflammatory cytokines augmented the regeneration in peripheral nerve crush injury. The immunohistochemical results showed an accumulation of inflammatory cells in the injured nerve tissues (28.33 ± 0.95/0.05 mm^2^) (Figure 6A). The accumulation of inflammatory cells was not altered in the AFS group (27.83 ± 0.94/0.05 mm^2^) (p = 0.7) (Figure 6B), whereas it was markedly alleviated in the Natto (12 ± 1/0.05 mm^2^) (p < 0.001) (Fig. 6C) and AFS+ Natto (11 ± 0.8/0.05 mm^2^) (p < 0.001) (Figure 6D) groups. However, there was no significant alteration in the distribution of neutrophil deposits either with or without Natto treatment (Figure 6E-H).

Deposition of fibrin occurred in nerve crush injury, and clearance of fibrin was related to better nerve regeneration. Fibrin activated macrophage migration and up-regulated expression of IL-1 and TNF-α two pro-inflammatory cytokines which are involved in inflammatory axonal damage and demyelination in the peripheral nervous system. Sciatic nerve crush injury markedly increased the deposition of fibrin, and the increase was statistically reduced by Natto treatment alone or the combination of AFS+ Natto (Figure 6K). On the other hand, crush injury triggered the production of inflammatory cytokines, including, IL-1 and TNF-α. The elevated production of TNF-α and IL-1β was attenuated in the Natto and AFS+ Natto groups (Figure 6J). Inducible inflammatory cytokines were not abrogated by AFS transplantation alone. The findings indicate that Natto, but not AFS, possesses an anti-inflammatory effect.

## Discussion

Either AFS or Natto treatment alone significantly reduced the neurological deficits, and the combined therapies had the most beneficial effect. The addition of Natto further prevented the AFS from apoptosis by inhibiting the fibrin deposition, which paralleled the suppression of macrophage aggregation and pro-inflammatory cytokines expression.

The mechanism of AFS involved in nerve regeneration has not been completely elucidated. Several postulations exist, including immunomodulation, secretion of neurotrophic factors or integration as part of host nerve tissue [2,3,23]. In our previous study, high expressions of mRNA, such as GDNF, BDNF, CNTF and NT-3, were found in amniotic fluid mesenchymal stem cells. Expressions of NT-3 and CNTF were demonstrated in transplanted AFS as well as in the nerve tissue [2]. The increased AFS survival either treated with G-CSF or Hyperbaric oxygen was in line with significant improvement in neurological function [8,9]. Furthermore, there was no evidence of S-100 positive cells in transplanted cells or alteration of immune cells adjacent to the crushed nerve (data not shown). Hence, the paracrine effect of AFS, which contributed to nerve regeneration, was regarded as the most likely mechanism.

After sciatic nerve injury, fibrin is deposited at the nerve, and its deposition exacerbates nerve damage [10]. Fibrin deposited in the sciatic nerve after injury changes the composition of extracellular matrix, inhibits Schwann cell migration, and induces pro-inflammatory cytokine expression [11,12]. Furthermore, deposition of fibrin induces macrophage accumulation [16]. In our previous study, oral administration of Natto attenuated the fibrin deposits in the crushed nerve and was correlated with the suppression of inflammatory cytokines expression and restoration of the blood-nerve barrier [21]. In the present investigation, the down-regulation of fibrin deposits not only aborted the macrophage migration but also attenuated the expression of inflammatory cytokines. The modulation of inflammatory cell deposits and the associated inflammatory cytokines paralleled the neurobehavioral improvement. This result further confirmed that Natto has the potential to inhibit fibrin deposits and pro-inflammatory cytokines.

The continuous survival and successful integration of implanted cells are regulated by multiple factors. There are several possible reasons for the short-term survival of implanted cells, including the detrimental effect of inflammatory cytokines, inadequate niches, abnormal apoptosis, and other unidentified mechanisms [24-26]. The modulation of monocyte-macrophage recruitment with the corresponding inflammatory cytokines either by G-CSF or hyperbaric oxygen significantly attenuated AFS apoptosis when AFS were grafted into the injured nerve. The percentage of the cell survival was in line with the significant nerve regeneration [8,9]. In the present investigation, Natto triggered the down-regulation of fibrin and inflammatory cytokines as well as the corresponding macrophage migration, and these events rescued AFS from apoptosis. The modulation of inflammatory response by Natto may be a potential adjuvant treatment in stem cell transplantation.

Nerve injury initiates inflammatory response and induces expression of pro-inflammatory cytokines such as TNF-α, IL-1β, and IFN-γ [27,28]. Inflammatory cells and inflammatory mediators not only cause tissue damage and secondary injury but also play a role in the regenerative process. In consideration of cell transplantation, an alternative role of inflammatory cytokines is to be an important determinant for the survival and fate of implanted cells. In a previous study, a significant accumulation of inflammatory cells was detected in the injured sites after nerve crush injury. The injured nerve tissues produced elevated levels of pro-inflammatory cytokines, including TNF-α, IL-1β, IL-6, and IFN-γ. Attenuations of inflammatory cytokines and associated inflammatory cells paralleled the decreased apoptosis of transplanted stem cells in nerve crush injury [2]. However, the expression of inflammatory cytokines was a mandatory response to nerve crush injury. In the early stage, the production of inflammatory cytokines was detrimental to nerve regeneration. In the late stage, the inflammatory cytokines were crucial in the recruitment of immature Schwann cells and the accelerated rapid migration of Schwann cells [29]. It is argued that suppression of inflammatory cytokines may hinder nerve regeneration in the late stage. Even though the inflammatory cytokines were suppressed, a significant amount of them were still retrieved from the crush site, which may be enough to support nerve regeneration in the late stage [8]. In this study, decreased transplanted cell apoptosis was in line with decreased inflammatory cell deposits and pro-inflammatory cytokines. Thus, the decrease in transplanted AFS apoptosis was due to the anti-inflammatory effect of Natto, which decreased inflammatory cell deposits and subsequently inhibited the secretion of inflammatory cytokines.

During nerve regeneration, the myelination of nerve fibers was a marker to determine the intensity of nerve regeneration [30]. Neurofilament expression indicated the early evidence of nerve regeneration potential [31]. The amount of S-100 immunoreactivity in myelinated fibers appeared to correlate directly with the thickness of the myelin sheath formed by Schwann cells [32]. Further, vacuole formation and vascular staining reflected the intensity of nerve regeneration [8]. Hence, in this study, we utilized neurofilament as an early marker and the expression of immunoreactivity of S-100 and vacuole counts as late markers to evaluate the intensity of nerve regeneration. Based on the expression of nerve regeneration markers, neurobehavioral studies, and electrophysiological parameter alteration, we found that either Natto or AFS transplantation alone had a similar effect but the combined treatment showed the most beneficial results.

Spontaneous recovery of motor function in a sciatic nerve crush injury model was reported several decades ago and again recently [30,33]. It could be argued that in these studies there was a tendency toward spontaneous recovery even without any treatment. If we had not used a serious nerve damage model such as nerve transection in the present study, the interpretation of the extent of nerve regeneration either by Natto, AFS, or the combined treatment would have amounted to sheer speculation. In our previous studies, nerve regeneration in a crush experimental model was not complete and serious neurological deficits remained at the time point of 4 weeks [2,8,9,21]. At the cut-off point of 4 weeks, there were significant differences between groups either in neurological deficits, electrophysiological parameters, or in nerve myelination which are all used as important indicators to evaluate any treatment of nerve regeneration. In the transection model, the suture technique and materials regarded as inflammation-provoking agents are used, and these factors may compromise the interpretation of the inflammatory response [8]. Thus, for the sake of simplicity, we used the crush model to investigate the effects of Natto on the survival of transplanted AFS in this study.

Hoechst 33342 has been used as an exogenous marker for cell transplantation. However, a major concern with the extrinsic labeling method is that the label could be diluted with cell division or could be redistributed from the transplanted cells to host cells [2,34,35]. A disproportionate number of Hoechst 33342 positive cells among the transplanted cells and host tissue may compromise the interpretation. The characteristics of this abnormal uptake by host tissue usually occurred either in the areas remote from or adjacent to the margin of the transplanted site. Furthermore, the expression of this abnormal signal occurred in the late stage (more than 2 weeks after AFS transplantation), and this signal was weaker than that in the transplanted cells. In this study, we did not clearly define what the percentage of Hoechst 33342 positive cells was in the AFS one week after transplantation. But we found that Hoechst 33342 positive cells were confined to the epineurial area without penetrating into the crushed nerve, and they expressed strong immunoreactivity without decaying, which implied that most of these cells were AFS. For tracking the fate of transplanted cells more precisely, endogenous markers such as anti-human nuclear antigen or anti-Y chromosome antigen are preferable.

## Conclusion

In the present study, the addition of Natto as adjuvant therapy in AFS transplantation rescued a significant amount of AFS from apoptosis, and this event escalated the nerve regeneration. Natto also inhibited the fibrin deposition, attenuated the macrophage migration, and down-regulated the expression of inflammatory cytokines. The modulation of the monocyte-macrophage system as well as inflammatory cytokines by Natto contributed to the reduced AFS apoptosis when AFS were grafted to the injured nerve.

## Competing interests

The authors declare that they have no competing interests.

## Authors' contributions

HC was in charged of all animal studies and surgery, participated in the animal model establishment and the first draft of the manuscript. DY carried out SNI animal model establishment. SP participated in the design of the study and performed the statistical analysis. ML and CJ participated in the quantitative analysis of TUNEL and determination of inflammatory cells and associated cytokines. SM contributed the culture technique of AFS and Natto extract administration. MH participated in the neurobehavioral evaluation. FC conceived of the study, and participated in its design and coordination. All authors read and approved the final manuscript.
